# Cross-sectional associations between screen time and the selected lifestyle behaviors in adolescents

**DOI:** 10.3389/fpubh.2022.932017

**Published:** 2022-09-27

**Authors:** Huiying Fan, Jin Yan, Zhen Yang, Kaixin Liang, Sitong Chen

**Affiliations:** ^1^School of Physical Education, Shanghai Normal University, Shanghai, China; ^2^Centre for Active Living and Learning, University of Newcastle, Callaghan, NSW, Australia; ^3^College of Human and Social Futures, University of Newcastle, Callaghan, NSW, Australia; ^4^Physical Activity, Sports and Health Research Group, Faculty of Movement and Rehabilitation Sciences, Katholieke Universiteit Leuven, Leuven, Belgium; ^5^School of Psychology, Shenzhen University, Shenzhen, China; ^6^Centre for Mental Health, Shenzhen University, Shenzhen, China

**Keywords:** television watching, video, computer, multiple lifestyle, Youth

## Abstract

**Background:**

In adolescents, excessive screen time leads to many adverse health outcomes and is associated with a variety of lifestyle behaviors. This study was conducted to investigate the associations between the two types of screen time and a variety of lifestyle behaviors in American adolescents.

**Methods:**

Based on the Youth Risk Behavior Surveillance System, this cross-sectional study was conducted. With the help of data collectors, participants self-reported screening time, lifestyle behaviors, and demographic data *via* well-validated tools.

**Results:**

19% and 43.4% of the participants spent more than two hours a day watching television and using the computer, respectively, while the prevalence of physical inactivity and insufficient sleep was 75.1 and 74.4% respectively. Furthermore, 11.9, 7.3, 14.3, and 21.3% of the participants reported skipping fruits, vegetables, breakfast and milk, respectively. Moreover, the prevalence of alcohol and tobacco consumption and sexual activity was 26.8, 5.3, and 23.5% respectively. More than two hours of television time was significantly associated with high risks of fewer frequency for eating fruit (OR = 1.605, 95%CI: 1.308–1.970), vegetables (OR = 1.389, 95%CI: 1.029–1.873), and smoking (OR = 1.465, 95%CI: 1.088–1.972). Computer/video time for more than two hours was significantly associated with high risks of physical inactivity (OR = 1.724, 95%CI: 1.531–1.941), insufficient sleep (OR = 1.354, 95%CI: 1.151–1.592), and not eating fruit (OR = 1.434, 95%CI: 1.179–1.745).

**Conclusion:**

Increased screen time may be associated with specific unhealthy lifestyle behaviors in adolescents. Furthermore, the associations between different types of screen time and various lifestyle behaviors varied.

## Introduction

Numerous studies have demonstrated that excessive screen time (ST) in adolescents is an independent factor of many adverse health outcomes, such as obesity, cardiovascular disease (1, 2), declined cognitive function and mental health disorders (3, 4). Adolescence is crucial lifetime periods, high ST is increasingly recognized as a serious public health problem and persists into early adulthood (5). Such evidence suggests that adolescents should limit their daily time exposed in front of screen-based devices for health promotion, with a maximum of ST of 2 h per day (6, 7). However, previous epidemiological studies reveal a concerning scenario that ST in adolescents is at a high level and continued to increase over the past decades (8, 9). This warns that adolescents' poorer health status is partly attributed to prolonged ST.

In addition to the adverse effects of excessive ST on health outcomes, it has been found that ST is associated with various lifestyle behaviors, such as smoking, alcohol use, physical activity, sleep and sexual risk behaviors (10–13). Nogueira et al. (14) reported that adolescents with more ST were more prone to engage in early sexual activity. Nelson and Gordon-Larsen (13) suggested that relative to physically active adolescents, those with high exposure to ST were more likely to smoke. Other studies have found significant associations of prolonged ST with undesirable levels of physical activity (PA) (15–17) and insufficient sleep (18). Furthermore, current evidence also indicates that prolonged ST is positively associated with poorer food consumption patterns and food preferences in adolescents (19–21), including lower intake of fruit, milk and vegetable (22–24), which in turn may result in unsatisfactory nutritional quality in adolescents (20, 25).

The noted studies have demonstrated the negative roles of ST on lifestyle behaviors, but those studies have some inherent some limitations, which leads to research gaps that should be addressed in the subsequent studies. One of the limitations is that previous studies rarely examined the different types of ST and their associations with lifestyle behaviors. Indeed, the associations between different types of ST and lifestyle behaviors would vary greatly owing to different underlying mechanisms. Exploring the different associations is beneficial to understanding the roles of ST in lifestyle behaviors promotion, which is also conducive to developing and implementing efficient interventions. Additionally, the previous research merely investigated a limited number of lifestyle behaviors in the correlational studies. However, different lifestyle behaviors are interrelated and co-dependent, which indicates the associations between ST and one specific lifestyle behavior can be estimated more accurately when controlling for other interrelated lifestyle behaviors. Even though, the extant research and evidence do not elucidate such associations.

This study aims to explore the associations between two types of ST and a variety of lifestyle behaviors in adolescents using the nationally representative sample of the United States.

## Method

### Study design and participants

Data was collected from a project of the Youth Risk Behavior Surveillance System (YRBSS) (26). This project was conducted biennially, and its designer was the Center for Disease Control (CDC), USA. The purpose of the YRBSS was to identify how the risk behaviors change as time goes by among the students in high schools. The survey of YRBS was performed upon approval of the Institutional Review Board of the CDC. There were three stages of sampling, and the investigator had surveyed 9–12 grade students from both public and private schools in the USA. The sampling frame of the first stage included 1,257 primary sampling units (PSUs). The 1,257 PSUs were divided into 16 strata. Fifty-four of these 1,257 PSUs were sampled and the probability was proportional to the general enrolment size of school for the PSU. As for the sampling of the second stage, the secondary sampling units (SSUs) included a physical school for students of grades 9 to 12 or a school that combined the schools nearby to provide the course of four grades. The data which was collected in 2019 included 13,872 students, and they were from 136 schools and invited to take the survey. Valid data was collected from 13,677 students for final analysis. For the YRBSS survey in 2019, the general rate of response was 60.3%, and it was worked out by multiplying the response rate of school (75.1%), and the response rate of students was 80.3%. The survey of YRBSS was performed after the students gave informed consent at the targeted schools, and their parents or legal guardians signed the form of consent. The students were instructed to give a response to the questionnaire which was computer-scannable with the help of trained data collectors. All the survey procedures were designed after ensuring that personal privacy is protected, and voluntary and anonymous participation was encouraged. Moreover, all study participants of were informed of the purpose of the research and the instructions were given prior to formal survey.

### Measures

#### Screen time

Participants answered the following two questions: “Averagely, how much time do you spend on watching TV, playing a video game or using the computer for purposes other than doing schoolwork at daytime?” (Including activities such as the Internet, computer games, Nintendo, Game Boy, PlayStation and Xbox),” with a paper-based questionnaire. Measures to assess ST have been confirmed with acceptable psychometric performance (27). The answers of these two measures were dichotomous to show whether the participants met the recommendation of spending <2 h of ST every day or failed to comply with the Canadian 24-h Movement Guidelines for Youth and Children (28, 29).

#### Lifestyle behaviors

In this study, the following lifestyle behavior was considered in the further analysis, including PA, nighty sleep, eating habits of fruit, vegetable, breakfast and milk, sexual behavior, alcohol use and current smoking status.

##### Physical activity

In this study, the single item question: “During the past 7 days, how many days were you physically active for a total of at least 60 min per day?”, which has been used to measure the physical activity of adolescents in previous research (30), was applied as a measurement of physical activity. Referring to previous studies, 0–6 day participation was defined as insufficient physical activity, while 7 day participation was defined as sufficient physical activity (30).

##### Sleep duration

Sleep duration was measured through a single item question: “On an average school night, how many hours of sleep do you get?”, which has been successfully applied to measure sleep duration in adolescents (31).

##### Fruit and vegetable intake

Fruit intake was assessed using a single item question: “During the last 7 days, how many times have you eaten fruit? (Do not count fruit juice.)”. While participants were required to answer whether they ate green salad, potatoes, carrots, or other vegetables during the past 7 days for vegetable intake measurement (32).

##### Eating breakfast and milk consumption

The participants were asked to answer whether they eating breakfast or how many glasses of milk they drunk during the past 7 days for the breakfast and milk consumption measurement (33).

##### Alcohol use and smoking

The alcohol and smoking were measured through single item questions, which have been applied in previous research (34), that how many days did participants have at least one drink of alcohol or smoke cigarettes during the past 30 days.

##### Sexual activity

Sexual activity was assessed by the question, that was ‘During the past 3 months, with how many people did you have sexual intercourse?’.

The measures of these variables can be found in Supplementary Table S1.

#### Covariates

Participants of the study were asked to report their demographic characteristics, such as race ethnic group (Hispanic/Latino, African American white or other race ethnic groups), grade, age (younger than 12 years or older than 18 years), and sex. Similarly, the participants were asked to report their weight and height through a paper questionnaire, so that the body mass index can be estimated to determine the state of obesity or overweight. For statistical analysis, the variables were treated as confounding variables.

### Statistical analysis

According to the YRBSS data analysis protocol, all the statistical analyses were performed based on the complex sampling design, to estimate nationally representative results. Missing data were not imputed in our study as the proportion was very small (Table 1). The statistical analysis was completed using SPSS 26.0. Because the variables included in the statistical analysis were categorical, descriptive statistics with percentages were presented to report the characteristics of the study sample. The weighted estimate of the variables was reported with a 95% confidence interval. To estimate the associations between different types of ST and the selected lifestyles behaviors, binary logistic regression was utilized, while controlling for sex, age, grade, race ethnic group, overweight and obesity. In the models, those reporting no for the outcome variable were considered the reference group, whereas the reference group for each independent variable was as follows: sufficient PA (yes), television watching hours (≤2 h per day), played video or computer games or used a computer (≤2 h per day), sleep duration was >8 h per night (yes), do not eat fruit (no), not eat vegetable (no), do not eat breakfast (no), do not drink milk (no), currently use alcohol (no), currently smoking (no), currently sexual activity (no). The odds ratio (OR) with 95%CI were calculated to assess the associations between different types of ST and lifestyle behaviors. The complex Sample Module of the SPSS was used to achieve the above-noted analysis. Statistical significance was set as *p* < 0.05.

**Table 1:** Demographic characteristics of the participants.

		***n***	**%**	**Weighted %**	**95%CI**	
**Total**		**13,677**	**100**	**/**	**/**	**/**
Sex						
	Female	6,885	50.3	49.4	47.9	50.9
	Male	6,641	48.6	50.6	49.1	52.1
	Missing	151	1.1			
Age group						
	≤ 12 years	60	0.4	0.3	0.2	0.5
	13 years	27	0.2	0.1	0.0	0.2
	14 years	1,699	12.4	11.9	10.9	13.0
	15 years	3,473	25.4	24.8	23.5	26.0
	16 years	3,628	26.5	25.6	24.5	26.7
	17 years	3,102	22.7	23.7	22.5	24.8
	≥18 years	1,616	11.8	13.7	12.6	14.9
	Missing	72	0.5			
Grade						
	9th	3,637	26.6	26.6	25.4	28.0
	10th	3,717	27.2	25.5	24.7	26.3
	11th	3,322	24.3	24.3	23.2	25.4
	12th	2,850	20.8	23.6	22.4	24.8
	Missing	151.0	1.1			
Ethnic group						
	White	6,668	48.8	51.2	46.4	56.0
	African American	2,040	14.9	12.2	10.2	14.6
	Hispanic/Latino	3,038	22.2	26.1	21.8	30.9
	All other ethnic groups	1,493	10.9	10.5	7.9	13.9
	Missing	438	3.2			
Overweight						
	Yes	1,933	14.1	16.10	14.90	17.50
	No	10,207	74.6	83.90	82.50	85.10
	Missing	1,537	11.2			
Obesity						
	Yes	1,795	13.1	15.50	13.80	17.30
	No	10,345	75.6	84.50	82.70	86.20
	Missing	1,537	11.2			

CI, confidence interval.

## Results

### Sample characteristics

The general characteristics of participants are presented in Tables 1, 2. The sample was almost evenly distributed by sex with 50.3% being females. Of the 13,677 adolescents, about 20% and more than 40% of participants failed to meet the ST guideline on television watching and using a computer, respectively. The prevalence of insufficient PA (75.1%) and short sleep duration (74.4%) was similar. In terms of eating habits, 11.9, 7.3, 14.3, and 21.3% of adolescents reported not eating fruit, vegetable, breakfast, and milk, respectively. On the other side, the proportion of current alcohol use and cigarette use was 26.8 and 5.3%, respectively. Furthermore, 23.6% of adolescents reported having sexual activity.

**Table 2:** Prevalence of screen time and lifestyle behaviors.

		***n***	**%**	**Weighted %**	**95%CI**	
**Total**		**13,677**	**100**	**/**	**/**	**/**
Television watching hours ≤2 h per day						
	Yes	10,200	74.6	80.2	78.7	81.7
	No	2,596	19.0	19.8	18.3	21.3
	Missing	881	6.4			
Played video or computer games or used a computer ≤2 h per day						
	Yes	7,246	53.0	53.9	52.1	55.6
	No	5,931	43.4	46.1	44.4	47.9
	Missing	500	3.7			
Sufficient physical activity						
	Yes	2,954	21.6	23.2	21.9	24.6
	No	10,266	75.1	76.8	75.4	78.1
	Missing	457	3.3			
Sleep for >8 h per night						
	Yes	2,930	21.4	22.1	20.6	23.7
	No	10,175	74.4	77.9	76.3	79.4
	Missing	572	4.2			
Do not eat fruit						
	Yes	1,631	11.9	11.9	10.5	13.4
	No	11,255	82.3	88.1	86.6	89.5
	Missing	791	5.8			
Do not eat vegetable						
	Yes	992	7.3	7.9	7.1	8.7
	No	10,765	78.7	92.1	91.3	92.9
	Missing	1,920	14.0			
Do not eat breakfast						
	Yes	1,956	14.3	16.7	15.3	18.1
	No	9,637	70.5	83.3	81.9	84.7
	Missing	2,084	15.2			
Do not drink milk						
	Yes	2,919	21.3	30.6	29.0	32.3
	No	6,570	48.0	69.4	67.7	71.0
	Missing	4,188	30.6			
Currently alcohol use						
	Yes	3,669	26.8	29.2	27.3	31.2
	No	8,942	65.4	70.8	68.8	72.7
	Missing	1,066	7.8			
Currently smoking						
	Yes	726	5.3	6.0	5.0	7.2
	No	11,591	84.7	94.0	92.8	95.0
	Missing	1,360	9.9			
Currently sexual activity						
	Yes	3,669	23.6	27.4	25.2	29.8
	No	8,942	63.9	72.6	70.2	74.8
	Missing	1,066	12.6			

CI, confidence interval.

### Associations between different types of ST and other lifestyle behaviors

Results of the logistic regression are shown in Table 3. Television watching for more than 2 h was significantly associated with higher odds of not eating fruit [OR = 1.605 (1.308, 1.970)], not eating vegetable [OR = 1.389 (1.029, 1.873)], and smoking [OR = 1.465 (1.088, 1.972)]. Furthermore, playing video games or using a computer for more than 2 h was significantly related to higher risks of insufficient physical activity [OR = 1.724 (1.531, 1.941)], sleeping for >8 h nightly [OR = 1.354 (1.151, 1.592)], as well as not eating fruit [OR = 1.434 (1.179, 1.745)].

**Table 3:** Associations between with screen time and the selected lifestyle behaviors.

	**Watched television for more than 2 h per day**			**Played video or computer games or used a computer for more than 2 h per day**		
	**OR**	**95%CI**		**OR**	**95%CI**	
Physical activity (REF = sufficient)	1.069	0.838	1.365	**1.724**	**1.531**	**1.941**
Sleep more than 8 h nightly (REF = yes)	0.948	0.814	1.104	**1.354**	**1.151**	**1.592**
Do not eat fruit (REF = no)	**1.605**	**1.308**	**1.970**	**1.434**	**1.179**	**1.745**
Do not eat vegetable (REF = no)	**1.389**	**1.029**	**1.873**	0.993	0.815	1.211
Do not eat breakfast (REF = no)	1.107	0.801	1.529	1.100	0.942	1.286
Do not drink milk (REF = no)	1.015	0.857	1.201	1.033	0.897	1.190
Currently alcohol use (REF = no)	1.124	0.968	1.306	1.056	0.900	1.240
Currently smoking (REF = no)	**1.465**	**1.088**	**1.972**	0.958	0.681	1.348
Currently sexual behavior (REF = no)	0.943	0.782	1.137	1.114	0.970	1.279

REF, reference group.

All models were controlled for sex, age, grade, ethnic group, overweight and obesity; Bold numbers: statistically significant at p < 0.05.

## Discussion

This study aimed to explore the associations of two types of ST (e.g., television viewing and video/computer game time) with some selected lifestyles behaviors in adolescents, using a nationally representative sample of US adolescents, based on the YRBSS data. The research findings of this study showed that more than 2 h of television viewing was associated with lower fruit intake, vegetable intake and higher frequency of smoking, while more than 2 h of video/computer games time were positively associated with insufficient PA, inadequate nocturnal sleep (<8 h per day) and lower fruit intake.

Across the previous studies, most of them focused on overall ST or television watching time instead of distinguishing different types of ST when examining the associations between ST and lifestyle behaviors (11, 35). While television viewing remains the most common type of screen-based activities among adolescents, video and computer games have also become part of everyday life for adolescents, television and video/computer games are also the most commonly studied types in screen research (36). Therefore, more research is needed to explore the associations between different types of ST and lifestyle behaviors, in order to understand how to promote healthy lifestyles from the perspective of limiting specifically diverse ST. Previous studies have reported that different types of ST are associated with different indicators of lifestyle (18, 37, 38). These findings can support our study, which television watching and video/computer game time linked with particular multiple lifestyle behaviors (e.g., physical activity, sleep). A possible explanation is that the effects of different types of ST might relate to time spent screen, content on screen, or even the context in which delivers various contents (35). For example, experimental studies have showed that watching television during meals may distract attention, leading to a delay in normal meal satiety times and a reduction in internal satiety signals (39), video and computer games are unlikely to have the same effect. Gebremariam et al. (40) found that television viewing was related to more exposure to advertisements for unhealthy foods/drinks content than computer use, and passive overconsumption of foods/drinks was more likely to occur during television viewing than computer use, especially when the latter involves gaming activities. In our study, more television watching was significantly associated with lower vegetable intake and fruit intake. This is consistent with previous research (24, 41). For example, Ramos et al. (42) found that television viewing was associated with a lower consumption of fruits and vegetables in European adolescents, this finding can also be supported, which collectively corroborate the present study (43). Several causal hypotheses have been proposed to explain why eating behavior would be inversely associated with television watching. One assumes that food advertising delivered by television watching affects adolescents' food selection (44–46), which makes children prefer unhealthy eating habits. In addition to this, the present study suggested that more than 2 h of television watching was associated with higher odds for smoking. Concerning the association between smoking and television watching, other studies reported findings similar to the current study. For instance, Thomas et al. found that television time increased the share of smokers and 16- to 21-year-olds adolescent smokers were particularly influenced by television (47). There are some plausible explanations for the associations between smoking and television watching. Contents from television about tobacco enable to normalize smoking behavior, such as standard advertisements, product placement, non-sponsored smoking by stars (47). Research also reported that television media coverage may contribute to public misconceptions regarding the associated health risks of smoking (48) and this misunderstanding would trigger adolescents' unintended smoking. Therefore, strengthening the censorship of tobacco-related content on the TV side may be necessary to reduce adolescents' behavior.

The current study found that sufficient PA was negatively associated with video/computer games time. This result is consistent with previous studies showing a negative correlation with video time and sufficient PA (41, 49). Xie et al. (15) proposed that the relationship between PA and the use of screen-based behaviors was complicated and may cause different results, and ST may affect PA through multiple mechanisms in opposite directions (15). One of the hypotheses is that ST have been to influence PA through displacing time that could be used for physical activity. Time displacement hypothesized when adolescents spent more time on video/computer games, they may have less time available for PA (50), which is supported by Sandercock (49), and Biddle (51). Moreover, longer durations of ST are associated with decreased physical fitness (52), and decreased muscle strength (39), which may also affects the level of PA. Furthermore, the high prevalence of insufficient physical activity might influence this association.

Insufficient sleep is a frequently occurred health issue among adolescents and an important public health issue (53). The 2011 Sleep in America Poll reported that about 60% of adolescents in the United States had <8 h of sleep on school nights (54). Concerning sleep, our finding was consistent with previous studies (55, 56) which found that insufficient sleep was associated with video/computer games time. Time displacement hypothesis also can explain this finding (57). Having more time spent in playing video or computer games, adolescents might have less time available for sleep. Moreover, psychological and physiological arousal owing to the computer use and the associated social interaction may also interfere with the timing to fall and stay asleep (18).

This study is with both strengths and limitations. In terms of study strengths, there is a large sample size that can increase the generalizability of research findings (only in the US population) and specific kinds of ST to be assessed associations with selected lifestyle behaviors. However, some study limitations must be acknowledged. This study is based on self-report and may have some reporting bias, such as adolescents may under-report the weight and over-report the height resulting in inaccurate BMI. And this study only focuses on traditional screen devices (TV, video and computer games) and does not include new generation screen devices (Smartphones and tablets), future studies should address comprehensively for more kinds of screen activities. Moreover, this study did not compare different adolescent populations (e.g., sex) and time segments (e.g., weekdays and weekends), which should be the direction of future research. Due to the limitations of the screen time measurement, this study did not include the measurements of the content of ST, which may confound results. Furthermore, ST and lifestyle behaviors are dichotomous variables in this study, which may affect the precision of the results. Accordingly, future studies should measure the content of ST and lifestyle behaviors in more details. Additionally, the survey for this study was conducted before COVID-19, and given the dramatic changes brought about by the pandemic, the results of this study need to be considered carefully in the relevant contexts. Meanwhile, this study was conducted in the United States, so the generalization of evidence needs more cautions.

## Conclusion

This study found that limiting screen time was positively associated with healthy lifestyle behaviors in adolescents. Of note, however, the associations between different types of screen time and various lifestyle behaviors varied. This study also offers practical implications on how to promote lifestyle behaviors in adolescents.

## Data availability statement

The data presented in this study are available on request from https://www.cdc.gov/healthyyouth/data/yrbs/index.htm (accessed on January 31, 2022).

## Ethics statement

Ethical review and approval were not required for the study on human participants in accordance with the local legislation and institutional requirements. Written informed consent to participate in this study was provided by the participants' legal guardian/next of kin.

## Author contributions

HF and JY: conceptualization. ZY: methodology, formal analysis, investigation, and writing—original draft preparation. KL: validation. SC: resources, supervision, and project administration. All authors: writing—review and editing. All authors contributed to the article and approved the submitted version.

## Conflict of interest

The authors declare that the research was conducted in the absence of any commercial or financial relationships that could be construed as a potential conflict of interest.

## Publisher's note

All claims expressed in this article are solely those of the authors and do not necessarily represent those of their affiliated organizations, or those of the publisher, the editors and the reviewers. Any product that may be evaluated in this article, or claim that may be made by its manufacturer, is not guaranteed or endorsed by the publisher.
