# Supplementary material for: Cross-sectional associations between screen time and the selected lifestyle behaviors in adolescents
Source: Front Public Health. 2022 Sep 27;10:932017. doi: 10.3389/fpubh.2022.932017 (PMC9552832; doi:10.3389/fpubh.2022.932017)
Supplement: Supplementary file 1 [file Table_1.docx]

**Table S1. Measures of independents of this study.**

| **Variable** | **Question** | **Response** | **Code** |
| --- | --- | --- | --- |
| Physical activity | During the past 7 days, on how many days were you physically active for a total of at least 60 minutes per day? (Add up all the time you spent in any kind of physical activity that increased your heart rate and made you breathe hard some of the time.) | A. 0 days B. 1 day C. 2 days D. 3 days E. 4 days F. 5 days G. 6 days H. 7 days | 7 days = sufficient physical activity  0-6 days = insufficient physical activity |
| Television viewing | On an average school day, how many hours do you watch TV? | A. I do not watch TV on an average school day B. Less than 1 hour per day C. 1 hour per day D. 2 hours per day E. 3 hours per day F. 4 hours per day G. 5 or more hours per day | 2 hours or less = limited television viewing  More than 2 hours = excessive television viewing |
| Video or computer games | On an average school day, how many hours do you play video or computer games or use a computer for something that is not schoolwork? (Count time spent playing games, watching videos, texting, or using social media on your smartphone, computer, Xbox, PlayStation, iPad, or other tablet.) | A. I do not watch TV on an average school day B. Less than 1 hour per day C. 1 hour per day D. 2 hours per day E. 3 hours per day F. 4 hours per day G. 5 or more hours per day | 2 hours or less = limited video or computer games  More than 2 hours = excessive video or computer games |
| Sleep duration | On an average school night, how many hours of sleep do you get? | 1. 4 or less hours B. 5 hours C. 6 hours D. 7 hours E. 8 hours F. 9 hours G. 10 or more hours | 8 hours or more = adequate sleep duration  Less 8 hours = inadequate sleep duration |
| Fruit intake | During the past 7 days, how many times did you eat fruit? (Do not count fruit juice.) | A. I did not eat fruit during the past 7 days B. 1 to 3 times during the past 7 days C. 4 to 6 times during the past 7 days D. 1 time per day E. 2 times per day F. 3 times per day G. 4 or more times per day | I did not eat fruit during the past 7 days = do not eat fruit  All the others = eat fruit |
| Vegetable intake | During the past 7 days, how many times did you eat green salad? | A. I did not eat other vegetables during the past 7 days B. 1 to 3 times during the past 7 days C. 4 to 6 times during the past 7 days D. 1 time per day E. 2 times per day F. 3 times per day G. 4 or more times per day | Choose any A of the 4 questions = do not eat vegetable  All the others = eat vegetable |
|  | During the past 7 days, how many times did you eat potatoes? (Do not count french fries, fried potatoes, or potato chips.) |  |  |
|  | During the past 7 days, how many times did you eat carrots? |  |  |
|  | During the past 7 days, how many times did you eat other vegetables? (Do not count green salad, potatoes, or carrots.) |  |  |
| Eating breakfast | During the past 7 days, on how many days did you eat breakfast? | A. 0 days B. 1 day C. 2 days D. 3 days E. 4 days F. 5 days G. 6 days H. 7 days | 7 days = do eat breakfast  all the others do not eat breakfast |
| Milk consumption | During the past 7 days, how many glasses of milk did you drink? (Count the milk you drank in a glass or cup, from a carton, or with cereal. Count the half pint of milk served at school as equal to one glass.) | A. I did not drink milk during the past 7 days B. 1 to 3 glasses during the past 7 days C. 4 to 6 glasses during the past 7 days D. 1 glass per day E. 2 glasses per day F. 3 glasses per day G. 4 or more glasses per day | I did not drink milk during the past 7 days = do not drink milk  All the others = drink milk |
| Alcohol use | During the past 30 days, on how many days did you have at least one drink of alcohol? | A. 0 days B. 1 or 2 days C. 3 to 5 days D. 6 to 9 days E. 10 to 19 days F. 20 to 29 days G. All 30 days | 0 days = do not use alcohol or smoking  All the others = alcohol use or smoking |
| Smoking | During the past 30 days, on how many days did you smoke cigarettes? | A. 0 days B. 1 or 2 days C. 3 to 5 days D. 6 to 9 days E. 10 to 19 days F. 20 to 29 days G. All 30 days |  |
| Sexual activity | During the past 3 months, with how many people did you have sexual intercourse? | A. I have never had sexual intercourse  B. I have had sexual intercourse, but not during the past 3 months  C. 1 person  D. 2 people  E. 3 people  F. 4 people  G. 5 people  H. 6 or more people | Responses of A and B = No  All the others = Yes |
